# Effect of Er,Cr:YSGG laser with a side-firing tip on decontamination of titanium disc surface: an in vitro and in vivo study

**DOI:** 10.1186/s40729-023-00469-z

**Published:** 2023-04-17

**Authors:** Lucia Kottmann, Rene Franzen, Georg Conrads, Stefan Wolfart, Juliana Marotti

**Affiliations:** 1 Department of Prosthodontics and Biomaterials, Centre for Implantology, Medical Faculty, RWTH Aachen University, Aachen, NRW Germany; 2 AALZ Aachen Dental Laser Center, Aachen, NRW Germany; 3 Department of Operative Dentistry, Periodontology and Preventive Dentistry, Medical Faculty, RWTH Aachen University, Aachen, NRW Germany; 4 Division of Oral Microbiology and Immunology, Department of Operative Dentistry, Periodontology and Preventive Dentistry, Medical Faculty, RWTH Aachen University, Aachen, NRW Germany; 5 Department of Reconstructive Dentistry, University Center for Dental Medicine UZB, University of Basel, Mattenstrasse 40, 4058 Basel, Basel-Stadt Switzerland

**Keywords:** Laser, Peri-implantitis, Side-firing tip, Decontamination, Titanium disc, Biofilm

## Abstract

**Purpose:**

To evaluate the effectiveness of an erbium, chromium:yttrium–scandium–gallium–garnet (Er,Cr:YSGG) laser with side-firing tip in decontamination of titanium (Ti) disc.

**Methods:**

In the first test series, 29 Ti-discs were contaminated with *Staphylococcus aureus* and treated as follows: positive control (no treatment); Perioflow; Laser A (0.75 W, 100 Hz), Laser B (1.5 W, 30 Hz); Laser C (no radiation, 60% water); and Laser D (no radiation, 50% water). For bacterial quantification, colony forming units (CFU, vital cells only) and quantitative PCR (qPCR, vital and devital cells) were performed. In a second test series, 92 Ti-discs were used, contaminated with in vivo-grown biofilm and treated as follows: positive control (no treatment); Perioflow; Laser E (1.5 W, 30 Hz), and Laser F (no radiation, 50% water). Considering the different and unknown culture conditions, quantification of bacteria was performed by broad-spectrum bacterial qPCR only. Based on the assumption that all cells of an organism contain an equivalent complement of genetic information, genome equivalent (GE) determination ensured the detection of the different intact and semi-intact genomes, regardless of type of bacterial species and vitality, circumvent the inherent bias of cultures.

**Results:**

The GE values were significantly reduced by all interventions in both test series, compared to the positive control group (*p* < 0.001). In the first test series with* S. aureus* as model organism, Perioflow yielded a lower GE than the Laser groups A–D (all *p* < 0.025). The number of CFUs was significantly reduced in the intervention groups compared to the positive control (*p* < 0.001), except for Laser A (*p* = 0.157) and Laser D (*p* = 0.393). In the second test series, none of the pairwise comparisons of the intervention conditions showed a significant difference (Perioflow vs. Laser E: *p* = 0.732; Perioflow vs. Laser F: *p* = 0.590; Laser E vs. Laser F: *p* = 0.379).

**Conclusion:**

The Er,Cr:YSGG laser with side-firing tip and Perioflow were equally capable of effectively decontaminating a Ti-disc surface. It is assumed that the bacterial reduction was largely due to the mechanical effect of the air and water stream.

**Graphical Abstract:**

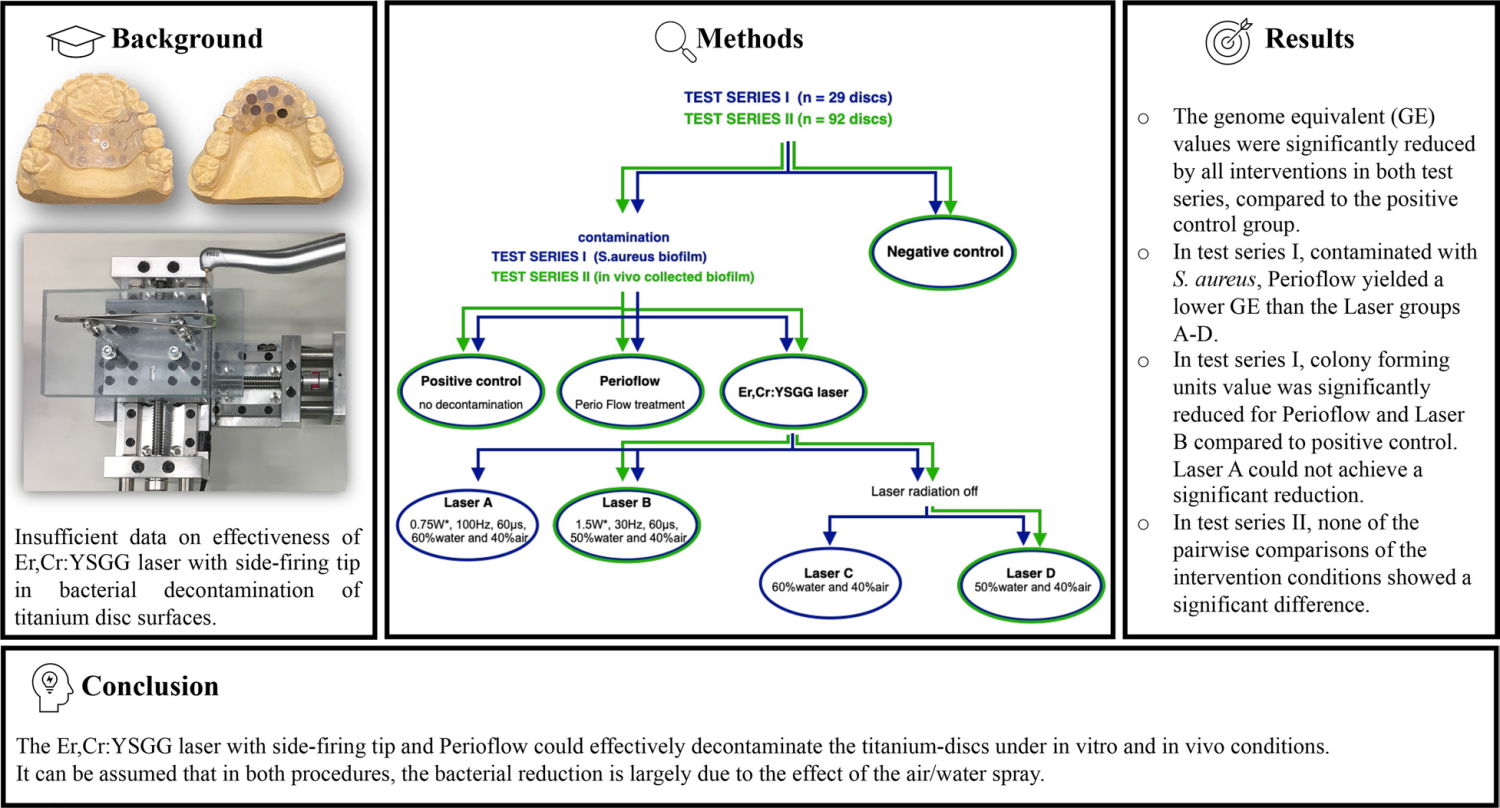

## Background

In addition to the implant properties themselves [1] and the method, timing [2] and biological/genetical conditions [3, 4] at implantation, biofilm formation is one of the most important etiological factors in the development of peri-implant infection [5, 6]. At the onset of infection, lesions are limited to the peri-implant soft tissue. This stage is referred as peri-mucositis [5, 7], which is reversible when early treatment is performed [5]. The histopathological and clinical conditions leading to the transition of peri-implant mucositis to peri-implantitis have not yet been fully elucidated. Sites affected by peri-implantitis show clinical signs of inflammation as well as an extension of the infection from the soft tissue to the peri-implant hard tissue, resulting in increased probing depths. The infection also progresses in a non-linear and accelerating pattern [6]. Peri-implant infections are characterized by increased numbers of numerous and diverse bacterial species, including *Porphyromonas gingivalis* and *Tannerella forsythia* [8]. In addition, increased opportunistic pathogens such as* Pseudomonas aeruginosa* and* Staphylococcus aureus* [9, 10] as well as increased occurrence of *Candida* were detected [11]. Peri-implantitis is defined, according to de Araújo Nobre et al., when there is more than 5 mm of probing depth, bleeding on probing and suppuration [12]. If left untreated, it can lead to further bone loss and, consequently, implant failure [13, 14]. According to a systematic review, the prevalence of peri-implant mucositis is reported to range from 19 to 65%, and the prevalence of peri-implantitis from 1 to 47%. Based on this, the estimated weighted mean prevalence for peri-implant mucositis and peri-implantitis are reported to be 43% and 22%, respectively [13].

Until the present moment, there is no consensus in the literature about the best treatment option for peri-implantitis [15]. What hampers treatment is the rough implant surface and the difficulty in accessing the affected area [16]. Several approaches for the decontamination of the affected implants are described: from non-surgical [17–20], to surgical procedures [21–23], using curettes [24], titanium brushes [25], ultrasonic devices or air-powder abrasion [20, 26], photodynamic therapy [27, 28], and high-power laser treatments [24, 29]. Promising results were described using erbium lasers [30–34], however, for better outcomes, a flap should be opened [16], considering that the laser tip must be used perpendicular to the surface.

The erbium, chromium:yttrium–scandium–gallium–garnet (Er,Cr:YSGG) laser operates at a wavelength of 2.78 µm and has the capability to be absorbed by water molecules and therefore results in minimal thermal damage to the surrounding tissues [35]. This physical property influences the ability of the wavelength to damage of the water-rich cells and provides the significant decontamination potential of this laser [36]. A side-firing tip (SFT) was developed for the Er,Cr:YSGG laser, aiming to access infected peri-implant sites without the need of opening flaps. In contrast to other tips, the SFT radiates at approximately a 90° angle and has a 180° directional handle. The manufacturer claims that the tip effectively removes more than 98% of biofilm from infected implants without damaging implant surfaces. The SFT could be a promising, minimally invasive treatment option for peri-implantitis. Studies showed that the SFT is well-suited for removing cement residues on implant surfaces [37], without causing surface alterations [38]. However, there is a lack of information in the literature about the efficacy of the SFT in removing biofilm of titanium surfaces. Thus, the aim of this study was to evaluate the decontaminating effect of an Er,Cr:YSGG laser, using a SFT, on machined titanium disc (Ti-disc) surfaces.

In addition to laser systems with appropriate tips, air polishing systems represent a promising non-surgical treatment method for cleaning implant surfaces [39, 40]. Regardless of some not finally clarified side effects (emphysema [41, 42], implant surface alteration [43, 44], changes in biocompatibility [45]), air polishing systems already find clinical application. Therefore, an air polishing system was chosen as the target device in this study.

The null hypothesis states that no difference can be seen between the treatment with an Er,Cr:YSGG laser with SFT compared to conventional treatment using air-powder abrasion spray.

## Methods

The protocol of this study was approved by the local Ethical Committee of the Medical School of RWTH Aachen University (Protocol Nr. EK 165/10) and was conducted in accordance with the Declaration of Helsinki and Good Clinical Practice.

Two series of experiments were conducted to evaluate the effectiveness of the Er,Cr:YSGG laser with SFT. Test series I corresponds to optimal laboratory conditions under which the model organism *Staphylococcus aureus* was subjected to standardized laser treatment. The conditions of test series II were approximated to a clinical environment, where a natural biofilm was subjected to manual laser treatment (see graphical abstract).

### Test series I

**Fig. 1:**
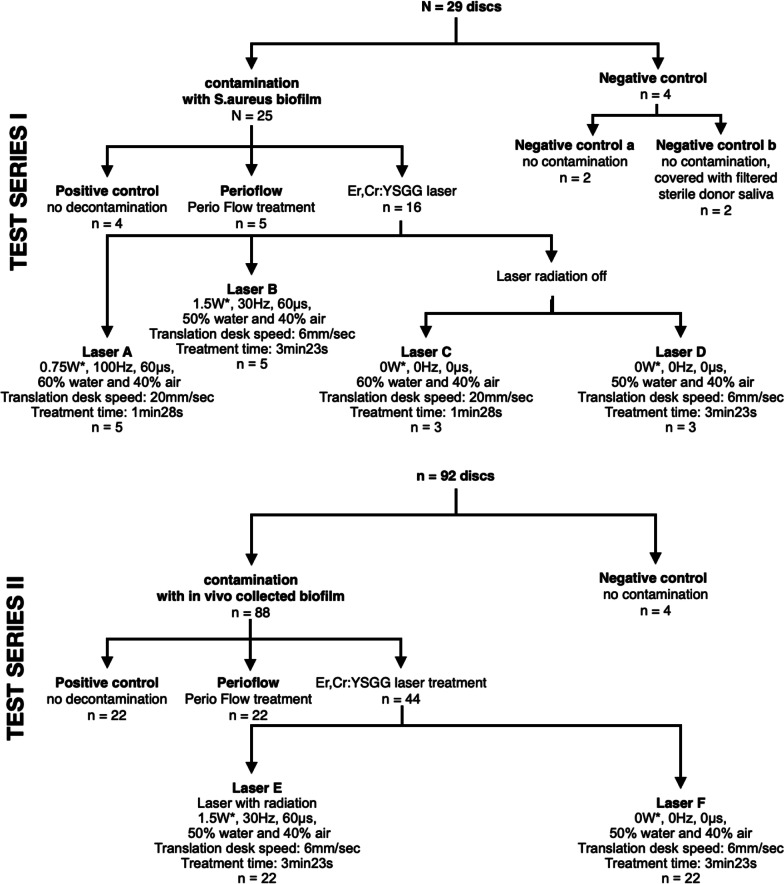
Groups distribution for the Test Series I and II. (Asterisk) The side firing tip’s calibration factor of 0.95 is not included

Twenty-nine machined Ti-discs (5 mm diameter, 1 mm thickness) provided by Straumann (Straumann, Basel, Switzerland) were used for this study and divided into 6 groups, as presented in Fig. 1 (Test Series I). The machined Ti-discs have a contact angle of 101.3 ± 3.3° and a roughness of S_a_ 0.118 ± 0.004 μm [46].

An *S. aureus* (ATCC 25923) standard suspension was prepared with 1.8 × 10E08 colony forming units (CFU)/ml. The ratio for choosing *S. aureus* as a model organism was (I) because of its general implication in implant-associated infections (oral and extra-oral); (II) the arsenal of adhesion molecules such as fibronectin and fibrin(ogen)-binding adhesins, which it shares with *P. gingivalis* and other oral pathobionts; and (III) practical reasons such as its robustness and survival on surfaces in time-consuming experiments. Therefore 1 μl of the initial suspension with an *S. aureus* concentration of 1.8 × 10E09 CFU/ml was applied together with 9 μl filtered sterile donor saliva on the surface of each sterile Ti-disc, which served as both pellicle and nutrient. The contaminated specimens were incubated for 24 h at 37 °C in a humid chamber to produce a defined biofilm. For this purpose, the discs were placed in wells of parafilm-sealed titer plates. Several empty wells were filled with sterile water to produce a humid atmosphere, optimal for biofilm growth. After 5 h, all discs were again inoculated with 5 μl filtered sterile donor saliva feeding the biofilm and preventing dehydration. The discs were stored on ice before and after all treatments to prevent further uncontrolled biofilm growth.

**Fig. 2:**
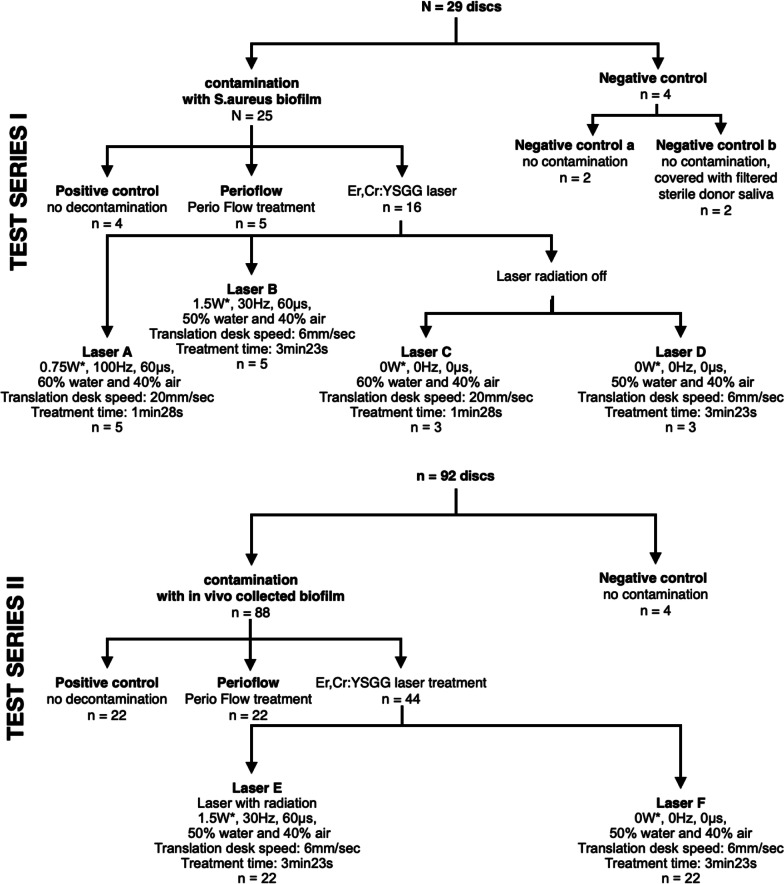
Translations desk with support for titanium discs and Er,Cr:YSGG laser with side-firing tip

A support was constructed to fix the Ti-discs throughout the experiment. This support stabilized the discs circularly and basally without making contact to the upper side (Fig. 2). Clamped into this fixture, the contaminated discs were ready to be air-polished or irradiated.

An Er,Cr:YSGG laser (Waterlase, Biolase, USA), operating at a wavelength of 2.78 µm in the infrared spectrum with a pulse duration of 60 μs in H-mode and 700 μs in S-mode was employed. A pulse frequency of up to 100 Hz and an average output power of up to 10 W can be adjusted. Laser energy is transported through a fiber optic system to the tip. Two parameter settings were selected. For the group Laser A, a fluence of 6 J/cm^2^ was sought and the parameters were set accordingly to 0.75 W average power, 7.5 mJ, 100 Hz, 60 μs, 60% water and 40% air spray. The parameters of the group Laser B complied with the manufacturer's recommendations: 1.5 W average power, 50 mJ, 30 Hz, 60 μs, 50% water and 40% air spray. Group Laser C is the corresponding control to group Laser A and Laser D to Group Laser B.

The SFT has a length of 18 mm and a fiber diameter of 800 µm. The maximal power of operation is specified to 2.25 W, a calibration factor of 0.95 must be included. This means an actual 0.71 W of average power is to be expected in the group Laser A at the fiber tip, and 1.43 W in the group Laser B. The average radius of a pulse at the recommended distance of 1 mm was determined under a microscope, where an average value of 209 µm was defined. This results in a beam cross-sectional area of 0.00136 cm^2^.

The support was mounted on a translation desk with linear XY axes (MOVTEC Wacht, Pforzheim, Germany), which allows a very precise and an exactly reproducible movement along these two axes (Fig. 2). The translation desk was programmed for this study to follow a grid pattern, to achieve a reproducible treatment of the Ti-discs. The position of the laser system did not change throughout the experiment. Special value was placed on avoiding unintended overlapping in areas where direction changes occurred. To ensure this, the grid covered an area of 1 cm^2^. In order to achieve a pulse overlap of 50% in the horizontal and vertical axis (pulse overlap of X axis is equal to pulse overlap of Y axis), the speed of the translation desk as well as the line spacing in the grid had to be adapted to the beam profile of the SFT. For this purpose, a line spacing of 200 μm was set for the pattern and the speed of the shift table was calculated as a function of the selected frequencies in the groups Laser A and Laser B. For Laser A, this resulted in a speed of 20 mm/s, and for Laser B 6 mm/s. The sum of total treatment time varied therefore between the groups: Laser A/Laser C 1 min and 28 s; Laser B/Laser D 3 min and 23 s. To prevent the spread of germs, the fixture, the translation desk, the area around it, and all components used throughout the experiment were kept—whenever possible—sterile and protected from outside contamination.

Air Flow Master Piezon (E.M.S. Electro Medical Systems, Switzerland), an air-abrasive polishing system, was used with distilled water and glycine powder (25 µm, Air-Flow Perio Powder, EMS). A single-use flexible plastic nozzle (length 18 mm) was fixed on a Perioflow-handpiece and was employed in a grid pattern by a single operator in non-contact mode, parallel to the surface of the Ti-discs for 30 s. Power and water spray volume was placed on the medium setting. This treatment is abbreviated as “Perioflow” henceforth.

In both trials, bacteria were measured by 16S-directed real-time quantitative PCR (qPCR) and the abundance reported as “genome equivalents” (GE). After each treatment, discs were placed individually into Eppendorf tubes, each with 2 glass beads and 250 μl bidest. All Eppendorf tubes were vortexed intensely to quantitatively remove the residual biofilm from the surface. A volume of 50 μl of the suspension was shock-frozen in liquid nitrogen and stored at − 70 °C as a live recovery sample. Of the remaining 200 μl, the DNA was isolated. For this purpose, the QIamp DNA Mini Kit was used and the appropriate Qiagen protocol (Qiagen, Venlo, Netherlands) was exactly followed but with using a mutanolysin–lysozyme solution to ensure complete lyses of all bacterial cells, including those with a thicker cell wall. Next, the bacterial cell number was determined via qPCR applying broad-spectrum (“universal” bacterial) primers, whereby the intact 16S gene of all bacteria (independent of viability) was detected. Each sample was subjected to three measurements. PCR protocols and details about standard curves and controls can be found in other publications of our group [1, 47].

Apart from the negative control, serial samples of all test specimens which had been shock-frozen and stored at − 70 °C after trial, were prepared with dilutions of NaCl. From each sample of the positive control, two times 20 μl of the dilution steps 1:100, 1:1000 and 1:10,000 were applied to blood agar plates (PB 5012A, Oxoid, Thermo Fisher Scientific, Hampshire, United Kingdom). For the Perioflow, Laser A, Laser B, Laser C and Laser D groups, the concentrated suspension and the 1:10 and 1:100 dilution levels were added to the blood agar plates using the same procedure. The negative control was applied only in concentrated suspension. All agar plates were incubated for 24 h at 37 °C in a humid chamber before the CFUs were counted. To obtain comparable values, the number of CFUs, counted three times, was multiplied by a factor of 50 to reach 1 ml, and then multiplied by the value of the respective dilution.

### Test series II

Ninety-two machined Ti-discs (Straumann, Switzerland) with same characteristics were used for Test series II. The Ti-discs were divided into 5 groups, as presented in Fig. 1 (Test Series II). The discs were distributed at random among the groups. On each trial day, 5–6 discs per group were treated.

**Fig. 3:**
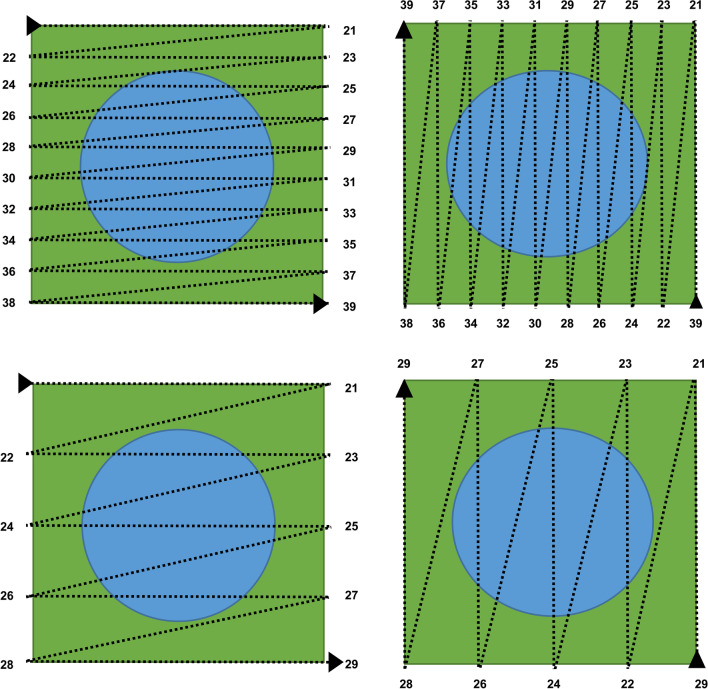
Splints for maxilla and mandible to collect in vivo-grown biofilm

For in vivo reproducible growth of biofilm on the Ti-discs, the discs were placed into bimaxillary splints intraorally for 16 h by a single test subject (Fig. 3). The discs were inserted into the recesses provided for this purpose, palatal to the splint, so that they were protected from the mechanical action of the tongue. The mandibular splint covered the sublingual area from tooth position 34–44, where unconscious tongue contact typically does not occur. Four trial days were planned to achieve the desired sample size of 22 discs per group. A uniform diet and the same oral hygiene measures were performed on all trial days.

**Fig. 4:**
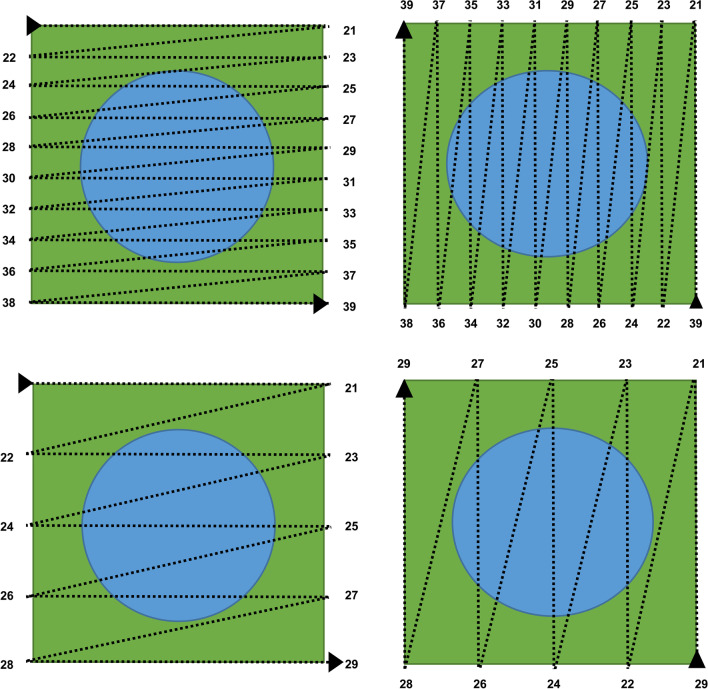
Schematic representation of the manually tracked pattern during laser irradiation (top) and perioflow treatment (bottom)

The laser and the SFT were used with the same material and methodology, as previously described. Based on the findings of Test series I and in accordance with the manufacturer's recommendations, only the settings of the groups Laser B and Laser D were included in Test series II, corresponding to the group Laser E (1.5 W, 30 Hz, 60 μs, 50% water, 40% air) and Laser F (50% water, 40% air). As described above, the SFT’s calibration factor must be considered. Irradiation was performed manually by a single, specialized operator. A grid pattern (Fig. 4) was maintained at a distance of 1 mm. The total irradiation time was 60 s per disc. The samples of the Perioflow group were treated for 20 s with the same material and methodology, as described above (Fig. 1).

The discs were kept on ice after removal from the trays, before, after and between all treatments. Subsequently, each disc was transferred to an Eppendorf tube filled with 250 μl bidest and 2 glass beads. These were shock-frozen in liquid nitrogen, stored at − 70 °C and later subjected to DNA extraction and qPCR. The Eppendorf tubes were thawed and vortexed intensely for at least 60 s each to remove the residual biofilm from the surface of each disc. 50 μl of the suspension were refrozen and stored at − 70 °C. Of the remaining 200 μl, DNA was isolated, and the cell number was determined via qPCR applying broad-spectrum bacterial primers, with the same material and methodology, as described above.

### Statistical analysis

The GE values from Test series I and II and CFU numbers from Test series I, were log-transformed to approximate a normal distribution. Of note, GE values still deviated from a normal distribution after log-transformation (Lilliefors–Kolmogorov–Smirnov test, *p* < 0.05), but we visually inspected quantile distributions of the raw data and the regression residuals (see below) and considered the approximation to be sufficiently close to a normal distribution to apply parametric statistical methods. The analyses using nonparametric methods (Kruskal–Wallis tests and Mann–Whitney *U* tests for pairwise comparisons) were repeated, with overall identical results.

Statistical analyses were performed using multilevel regression models with random intercepts per Ti-disk, to account for repeated measurements. Significance testing was carried out through *t*- and *F*-tests on model coefficients, using Satterthwaite approximations for degrees of freedom. Post hoc comparisons were conducted using pairwise contrasts. All analyses were performed in R 3.6 with the lme4 and lmerTest libraries.

To assert retest reliability between repeated measurements, the intraclass correlation coefficient (ICC) was calculated as the variance of the per-disc random intercept divided by the total random and residual variance. ICC values close to 1 indicate good retest reliability.

## Results

### Test series I

**Fig. 5:**
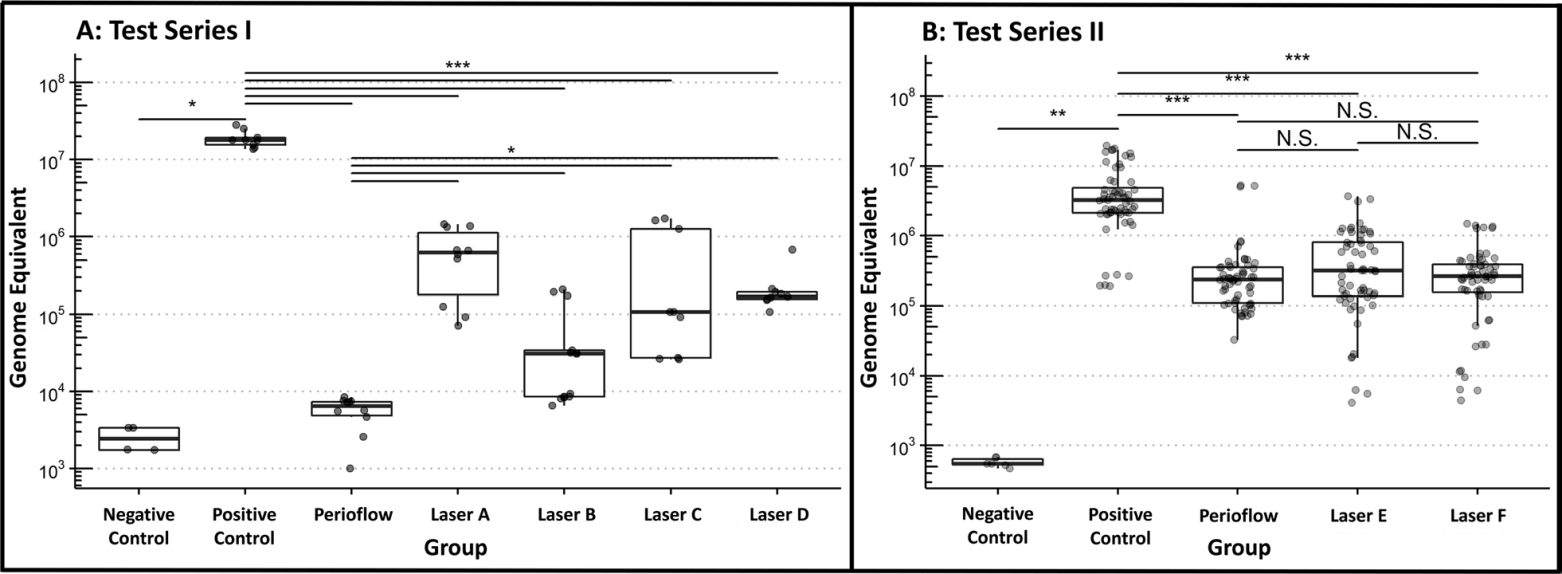
Genome equivalent values across all groups in the first (**A**) and second (**B**) test series. Dots represent single qPCR measurements (3 per disk)

The GE values of the positive control were 3 to 4 orders of magnitude higher compared to the negative control (*t*_5_ = 39.7, *p* < 0.001). GE values for all groups are shown in Fig. 5. Taken together, GE values in the five intervention groups were significantly reduced compared to the positive control (*F*_3,76_ = 20.4, *p* < 0.001). In pairwise comparisons, the GE value was significantly reduced in each intervention group compared to the positive control (*t* < − 4.8, *p* < 0.001). Furthermore, in pairwise comparisons, a higher GE value was found in Laser groups A–D compared to Perioflow (*t* > 2.4, *p* < 0.025).

**Fig. 6:**
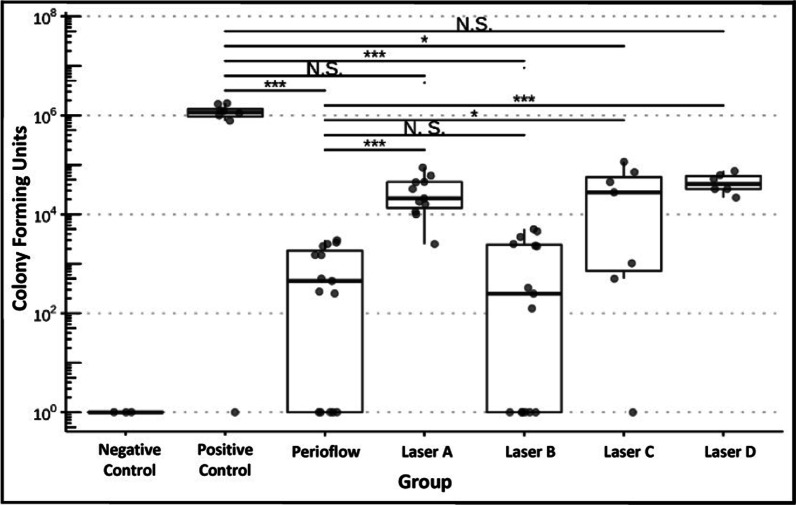
Number of CFUs (representing viable *Staphylococcus aureus* cells) across all groups in the first test series. Dots represent single CFU measurements (3 times counted per disk). Dots plotted at 10^0^ have an actual value of 0, not 1 (artificial correction)

CFU values for all groups are shown in Fig. 6. CFU values were significantly higher in the positive control group compared to the negative control (*p* = 0.037), and values for the five intervention groups were significantly reduced compared to the positive control (*F* = 10.6, *p* < 0.001). In pairwise comparisons, the CFU value was significantly reduced in the intervention groups compared to positive control (all *t* < − 5.2, all *p* < 0.001), except for Laser A (*t* = − 1.43, *p* = 0.157) and Laser D (*t* = − 0.86, *p* = 0.393). In pairwise comparisons between intervention groups, a higher CFU value was found in each group compared to Perioflow (*t* > 2.4, *p* < 0.025), except for Laser B (*t* = − 0.28, *p* = 0.784).

### Test series II

GE values in the positive control group were 3 to 4 orders of magnitude higher compared to the negative control (*t*_20_ = 10.4, *p* < 0.001) (see Fig. 5). Moreover, retest reliability was nearly perfect across all disks (ICC = 0.985). GE values for all groups (excluding the negative control) are shown in Fig. 5. Taken together, GE values for the three intervention groups differed significantly between conditions (*F*_3,76_ = 20.4, *p* < 0.001). In pairwise comparisons, the GE value was significantly reduced in each intervention group compared to the positive control (all *t* < − 5, all *p* < 0.001). However, none of the pairwise comparisons between the intervention conditions showed a significant difference (Perioflow vs. Laser E: *t*_76_ = 0.3, *p* = 0.732; Perioflow vs. Laser F: *t*_76_ = − 0.5 *p* = 0.590; Laser E vs. Laser F: *t*_76_ = 0.9, *p* = 0.379).

## Discussion

Significant bacterial reduction was achieved by both Perioflow and Er,Cr:YSGG laser treatment with SFT. The null hypothesis was confirmed, no significant difference was found between the two methods. Both the Er,Cr:YSGG laser with SFT and Perioflow were able to effectively decontaminate the Ti-discs. The decontaminating effect of Perioflow has already been demonstrated in other studies [26, 42]. Promising results were also shown for the Er,Cr:YSGG laser with different tips [24, 30, 38]. However, no studies have yet been conducted on the bactericidal effect of the Er,Cr:YSGG laser with SFT.

In Test series I, the group Laser B showed a significantly better bactericidal effect compared to the group Laser A. It should be noted that the settings of the group Laser A resulted from an equivalent calculation of the fluence of 6 J/cm^2^ advised in previous peri-implantitis studies on the new SFT, taking into account repetition rate, pulse overlap factor and the motor displacement speed of the translation desk. The goal here was specifically to compare the fluence of 6 J/cm^2^, not to achieve the highest possible bactericidal effect. With the settings of group Laser B, corresponding to the manufacturer's recommendation for peri-implantitis treatment with the SFT, effective bacterial reduction was achieved. Chegeni et al. showed that under those conditions no surface alterations should be expected [38].

Test series I shows a difference of about one order of magnitude between the results of CFU analysis and those of DNA extraction. This difference is mainly based on differing analytical methods. Genome equivalents (16S operons) are detected by means of qPCR [48]. Each *S. aureus* cell has five to six 16S operons [49], and one colony counted in CFU analysis is formed on average by at least two or (many) more cells (grape-like clusters). Taken together, both facts explain the difference by a factor of 10 between the results of CFU analysis and qPCR. Overall, it can also be observed that the results of Perioflow, Laser A and Laser B, even after applying the natural factor of 10, still show lower values in CFU analysis than in qPCR. This is based on the fact that only vital, culturable cells can be detected in CFU analysis [50], whereas qPCR counts 16S sequences, independent of cell-viability [48]. In the latter, thus also non- or poorly culturable cells, devitalized cells, cell segments, co-/aggregated cells, and DNA in matrix material is counted [51]. Both methods were used in Test series I to get the best overview and to reduce any influence from the duration of the test (e.g., natural dieback of cells).

In contrast to Test series I, Test series II was carried out with a heterogeneous in vivo-grown biofilm. Since this represents a very broad spectrum of microorganisms including very fastidious or even unculturable species, CFU counting was not chosen as method [52]. Here, applying 16S-directed qPCR and broad-spectrum primers (sometimes referred as “universal” but this term is surely an overstatement [47]), the detection of many different intact and semi-intact genomes, regardless of the type of bacterial species, is guaranteed [51], thus circumventing the inherent bias of cultures [53]. However, it warrants further consideration that part of the laser effect could be due to milder deactivation of bacteria. In this case, the DNA would not be completely destroyed and would remain measurable in qPCR. As a matter of fact, our molecular measurement procedure is at least partially blind to this portion. However, from the comparison of the results of CFU analysis and DNA extraction of Test series I it can be deduced that this proportion is rather low.

The results of Test series II confirmed the trend that emerged in Test series I: Perioflow and Laser E led to a significant reduction of bacterial load. A rather unexpected result was recorded by group Laser F, which performed just as well as Perioflow and Laser E. It seems likely that the disinfection by Er,Cr:YSGG laser measured in Test series I is mainly based on the ablative effect of the spray and not on the actual beam. This would mean that it was not the selected parameters that were decisive for the results of Test series I, but rather the treatment duration itself. The time factor was determined by working to achieve an optimal pulse overlap of 1. The time difference between the groups Laser A/Laser C and groups Laser B/Laser D was approximately 120 s. However, the fact that the groups Laser A and Laser B show better results than the correlated groups Laser C and Laser D warrants further consideration. The discrepancy between the results of the test series must therefore be based on the altered test environment, which affects the handling of the laser as well as the biofilm itself.

While an *S. aureus* monoculture was used in Test series I, a heterogeneous (thus natural and complex) biofilm was used in Test series II. As described in literature, the monoculture is an artificial circumstance, as microorganisms normally grow in complex ecological systems in natural habitats [54]. It is possible that as the complexity of the biofilm increases, the laser radiation can penetrate less deeply into the biofilm structure. Accordingly, the ablative effect of the air and water would then come to the fore.

In addition, in Test series I, an optimal laser treatment by means of a translation desk took place while the laser was operated manually in Test series II. The Laser’s beam profile has a diameter of approximately 209 μm, the corresponding profile of the spray is larger but the profile of the Perioflow device is still significantly larger. Despite very careful and replicable guidance of the laser handpiece, the size of the beam profile makes it impossible to achieve comparable surface coverage with manual handling than when relying on the translation desk. Therefore, the effect of the spray clearly dominates. This would mean that the results of the groups Laser B and Laser D are more or less exclusively due to the spray of the laser. The measurable effect of the laser radiation was minimized, and the groups Laser E, Laser F and Perioflow achieved almost identical results. A striking feature is the significantly greater scatter in the groups Laser E and Laser F compared to Perioflow. The different radiation profiles likely play a decisive role here as well. While the principle of Perioflow treatment is mainly based on an ablative effect and optimized for this purpose, the ablative effect of the spray of the laser is designed to be supportive. Although the results of this study do not suggest a significant improvement of the Perioflow treatment by a chemical effect of the glycine powder, it seems to provide an abrasive contribution that minimizes scattering. The small beam profile of the laser may not minimize scatter as effectively. It should be mentioned, that although the scatter in the groups Laser E and Laser F is larger, there are also clearly stronger outliers towards the bottom compared to Perioflow. In these cases, the manual laser treatment even achieved better surface coverage than the Perioflow treatment. Further studies with comparable control groups should be conducted to verify this suspicion.

Nevertheless, the effects of the water/air spray on the decontamination of the implant surface are a critical issue: on the one hand, bacteria washed away can continue to cause dysbiosis in the oral cavity if they have not been killed beforehand; on the other hand, the angulation of the water/air spray hitting the implant surface in both devices is not always ideal when applied to clinical peri-implantitis related defects. In these cases, the ablative effect of the air–water spray could be less important than under the conditions chosen here, which could also affect the success of the treatment.

However, treatment with Perioflow presents a risk of emphysema formation [41, 42]. In addition, the surface is altered in terms of roughness [43] and composition [44] in such a way that biocompatibility may decrease [45]. Laser can be used very precisely and selectively, and the associated spray is not damaging the surrounding tissue. In fact, significantly better healing can be expected after laser treatment [30, 55]. Yao et al. (2020) found that Er,Cr:YSGG laser-treated titanium surfaces show an increased viability and proliferation of fibroblasts as well as an increased differentiation of osteoblasts. They express the strong assumption that the treated surfaces directly stimulate the osteoblastic differentiation of fibroblasts. In addition, this study demonstrated that the adhesion of *P. gingivalis* and the formation of colonies on the Er,Cr:YSGG laser-treated titanium discs decreased in a time-dependent manner. The adhesion of *P. gingivalis* was even significantly lower on test days 1, 3 and 5 than in the control group [55].

When considering the results of this study, it should be noted that only flat Ti-discs were used, which correspond to an implant abutment in their microscopic and macroscopic properties. A mature biofilm in the threads of an implant is much more stable, as it can take advantage of the macroscopic conditions. Possibly, in this case, the ablative effect of the water/air spray would fade into the background and the effect of the laser radiation would become more evident due to the depth of penetration into the biofilm. In addition, it has already been demonstrated in other studies that depending on the material property initial biofilm formation, cleaning ability and restoration of biocompatibility are strongly influenced [46]. In this study, machined Ti-discs were used, which have a comparatively smooth and hydrophobic surface [46]. Other surface properties would probably also influence the results. In this case, it can also be assumed that rougher surfaces would lead to better bacterial adhesion and reduce the effect of the water/air spray, which would allow the laser effect to come to the fore. Further studies are needed to evaluate the effectiveness of the Er,Cr:YSGG laser with SFT on other surfaces.

The small sample size in Test series I is a limitation in this study. However, even with the small sample size, significant results were achieved, and further experiments would not have yielded any new findings. Therefore, it was decided to conduct Test series II, aiming at a larger sample size and a near-clinical treatment setting.

Another limitation of the study is the *Staphylococcus aureus* suspension which was chosen to contaminate the titanium discs in Test series I instead of a heterogeneous biofilm. The aim was to apply a precisely defined concentration of germs and thus confirm the precision of the evaluation methods before working with a heterogeneous biofilm in Test series II. In this way, a precise evaluation of CFUs was possible and it could be ensured that the CFU analysis would achieve a reliable result. These results could then in turn confirm the accuracy of the qPCR results. Thus, the source of error that the laser would not completely destroy the germs and thus large portions of DNA fragments would falsify the qPCR results could be disproved. In addition, *S. aureus* has already proven to be very robust in comparable studies [56] and it was possible to reliably measure the germ reduction through the effect of the laser / Perioflow, while taking into account the concern that the results would be falsified by the natural death of the germs in the course of an experimental day.

Furthermore, it should be emphasized that the angulation of the tip/nozzle to the implant surface is of crucial relevance. In this study, only an ideal angulation, recommended by the manufacturer, was used. In a clinical situation, such an approach certainly cannot be guaranteed. The change in effectiveness with deviations of angulation remains as a limitation of this study and requires further investigation.

## Conclusion

The Er,Cr:YSGG laser with side-firing tip and Perioflow could effectively decontaminate the titanium discs under the conditions of this study. It can be assumed that in both procedures, the bacterial reduction is largely due to the ablative effect of the air/water spray.

## Data Availability

The full data of this study are available upon request to the corresponding author.
